# Concomitant intra-atrial excision of the left atrial appendage during robotic-assisted minimally invasive cardiac surgery

**DOI:** 10.3389/fcvm.2023.1074777

**Published:** 2023-03-02

**Authors:** Mihnea Ghinescu, Ulrich F. W. Franke, Melisa Ortega, Franziska Hüther, Magdalena I. Rufa, Nora Göbel

**Affiliations:** Department of Cardiovascular Surgery, Robert-Bosch-Hospital, Stuttgart, Germany

**Keywords:** left atrial appendage occlusion, robotic-assisted cardiac surgery, atrial fibrillation surgery, thoracoscopic minimally invasive surgery, novel surgical technique

## Abstract

**Objective:**

Surgical closure of the left atrial appendage (LAA) in patients with atrial fibrillation undergoing cardiac surgery can decrease the risk of stroke and thromboembolism and should therefore be considered. In minimally invasive, thoracoscopic, or robotic-assisted mitral valve surgery, however, external procedures such as clip application or epicardial resection are not feasible due to anatomic limitations and the reduced size of the access port. Internal suture closing techniques bear the risk of recurrent LAA reperfusion, so far. We present a novel surgical technique of LAA excision and subsequent defect closure from the interior aspect of the atrium.

**Methods:**

We developed this novel technique during robotic-assisted cardiac surgeries. In short, the LAA is invaginated into the left atrium, excised completely at the base using scissors and the stump is then closed from the inside with a two-layer looped PTFE suture. We give a detailed step-by-step description of the technique.

**Results:**

A total of 20 patients received intra-atrial LAA excision so far. Complete resection of the LAA without any residual stump or bleeding was achieved in all cases. There were no procedure-related complications.

**Conclusion:**

The intra-atrial LAA excision technique shows promising preliminary results regarding efficacy, safety, and reproducibility during robotic-assisted cardiac operations and could be recommended for all right-sided minimally invasive cardiac surgical procedures.

## Introduction

Atrial fibrillation is a frequent comorbidity in patients suffering from valvular heart disease, especially in patients with mitral regurgitation. About 12% of patients presenting for cardiac surgery have concomitant atrial fibrillation carrying an elevated risk of thromboembolic complications, stroke and mortality (1, 2). The left atrial appendage is by far the most frequent location of emboli formation. Therefore, it has been addressed as a therapeutic target. Angiographically guided occlusion has been shown to effectively reduce the risk of stroke and thromboembolism, as has surgical LAA closure recently (3–5).

However, the optimal surgical technique of LAA closure has not yet been definitively established. Several techniques and devices are currently in use which all come with their specific limitations. A variety of mechanical devices are available (clip applicators, surgical staplers), as well as open surgical techniques (excision of the LAA and defect closure using mattress sutures, external purse string closure). These are, however, mainly applicable with full sternotomy access. Especially the purse string closure bears an elevated risk of residual pouch and thus blood flow, potentially significantly increasing the risk of thrombus formation (6, 7).

The past years have seen an increase in the use of minimally invasive surgical techniques in cardiac surgery, culminating in robotic-assisted heart surgery through right-sided mini-thoracotomy. Through 4 trocar ports and a very small intercostal incision, access can be gained to both atrioventricular valves and the corresponding atria, thus facilitating a wide array of mitral and tricuspid valve interventions but also atrial septal defect closure, Myxoma removal or atrial ablation procedures. The surgery of the LAA is, however, somewhat of a challenge, because it can hardly be accessed and visualized from the outside. Therefore, external techniques such as clipping or stapling are technically demanding. From the inside, purse string closure of the LAA is arguably as inefficient as its external variation. The running-suture double-layer closure is the only truly safe technique so far. We, therefore, developed this novel technique for safe and effective LAA closure during the last 2 years of experience with robotic-assisted cardiac surgery. The exact surgical technique is presented in a step-by-step description and experience with the first 20 patients is given.

After gaining access to the pericardium and establishing cardiopulmonary bypass, the main procedure is performed (mitral/ tricuspid valve repair, atrial septal defect closure, etc). In case of concomitant left atrial ablation the cryo-procedure is done first to ascertain completeness of lesion set. The opening of the LAA is then visualized. Through light traction using the robotic instruments the LAA is completely invaginated into the left atrium and resected along the demarcation line using scissors. All trabeculated tissue is removed. This leaves a defect in the lateral aspect of the left atrial wall, which is then closed using a double-layered stitch using a 5-0 PTFE suture. As seen in the photos, the edges of the defect are easily identifiable and can be very precisely manipulated without the risk of injuring neighboring structures, the most important of which is the circumflex branch of the left coronary artery.

We found that creating a loop allows for the use of a single suture and decreases the duration of the procedure without compromising safety in any way, as described before (8). The procedure only extends the operating time by around 10–15 min. In our series of 20 patients we haven't encountered any serious bleeding complications or residual stump of the left atrial appendage, as confirmed by intraoperative transesophageal echocardiography.

## Surgical technique

After placing the patient on the operating table in a supine position with slight elevation of the right hemithorax, the field is disinfected and covered with foil. Establishment of cardiopulmonary bypass is achieved through open femoral cannulation using a 2 cm incision. An additional superior vena cava cannula is inserted through the right internal jugular vein in order to optimize venous drainage and facilitate isolation of the right atrium (total bypass) in case it is needed. Four access ports are created for the robotic instruments (three on the middle axillary line roughly in intercostal spaces 2, 5, and 7 and one in the 4th intercostal space lateral to the sternum for the atrial retractor). Two additional access incisions are created for conventional surgical instruments, the cardioplegia cannula, and the aortic crossclamp, respectively. After gaining access to the pericardium and clear visualization of the atria and ascending aorta, the Chitwood aortic cross clamp is applied and cardioplegic solution is infused into the aortic root. Alternatively, the IntraClude system is used; a transfemoral combined arterial cannula, aortic balloon occlusion catheter and cardioplegia/ venting catheter, negating the need for an extra surgical incision and significantly reducing operation times. Following cardioplegia the left atrium is opened and the atrial retractor is placed in position. We usually perform the valve procedure first although this is not mandatory.

## Step-by-step

The complete procedure is summarized in Video 1 (Figures 1–10).

**Figure 1:**
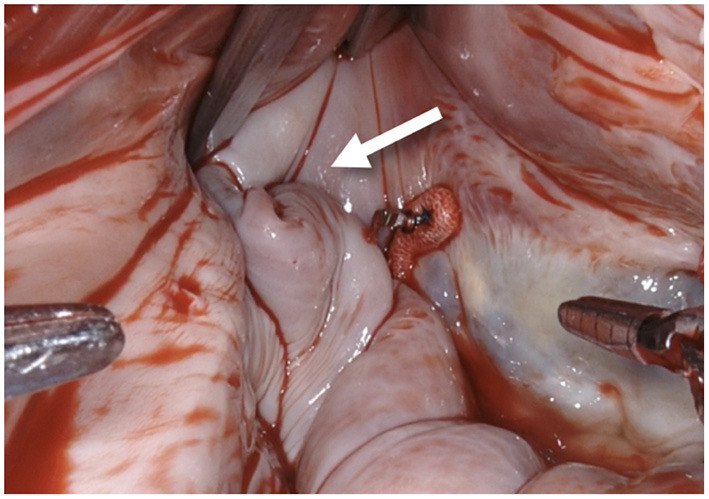
After performing a mitral valve repair, the entrance to the left atrial appendage (arrow) is exposed by using the atrial retractor, as seen on the upper edge.

**Figure 2:**
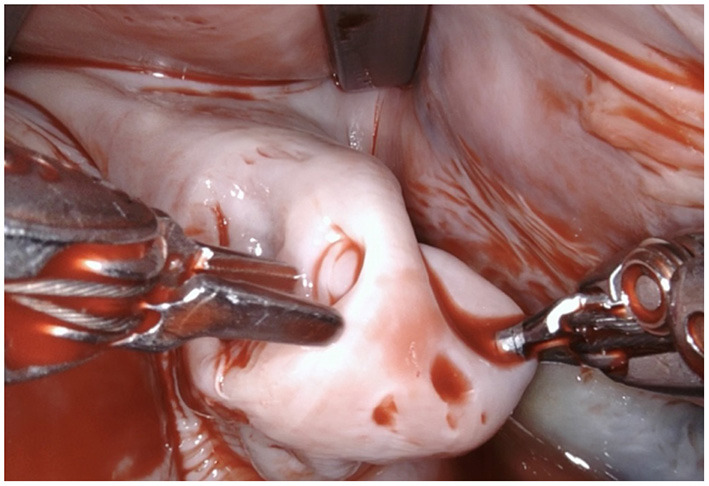
Using the two robotic instruments (left-hand: DeBakey forceps, right-hand: needle-driver) the left atrial appendage is invaginated into the left atrium by applying light traction until the rim of the base of the LAA is clearly identifiable. Care needs to be taken due to the fragility of the tissue and lack of feedback from the robotic instruments.

**Figure 3:**
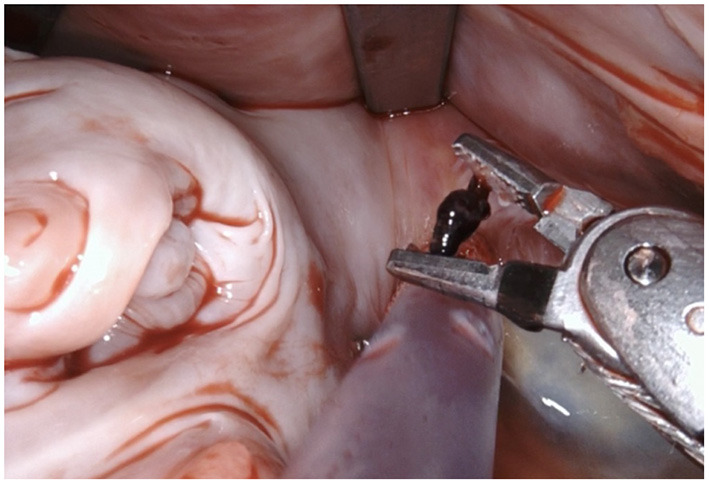
In this particular case a small thrombus was identified in the LAA and removed.

**Figure 4:**
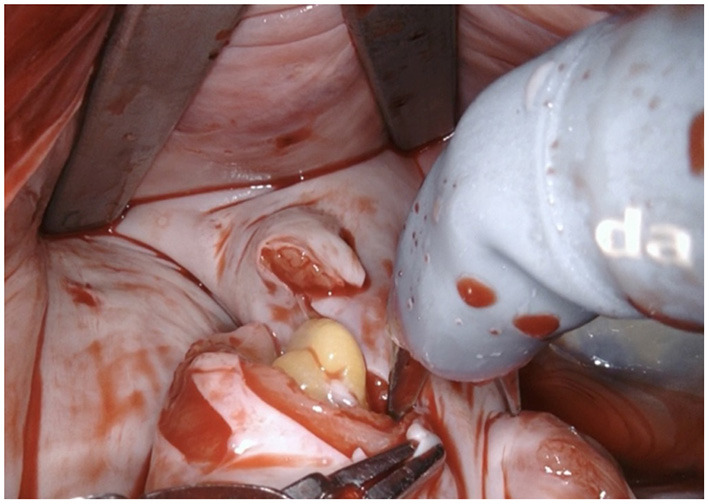
The LAA is completely resected at the base using bipolar scissors (right-hand). By light traction a basal rim demarcates serving for complete resection whilst saving nearby sensitive structures like the circumflex artery. Any stray tissue including pericardial fat that may make its way through the defect can be easily identified and removed.

**Figure 5:**
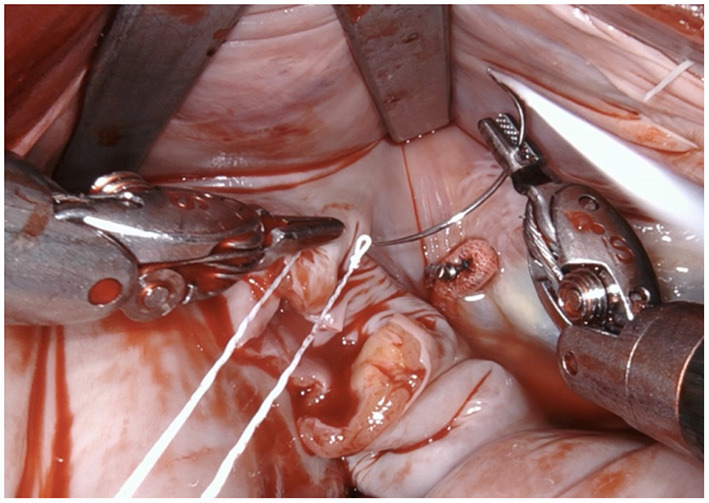
The resulting defect is sutured using a PTFE 5-0 suture using a double layer technique. We fashion a loop in the middle of the suture (see below).

**Figure 6:**
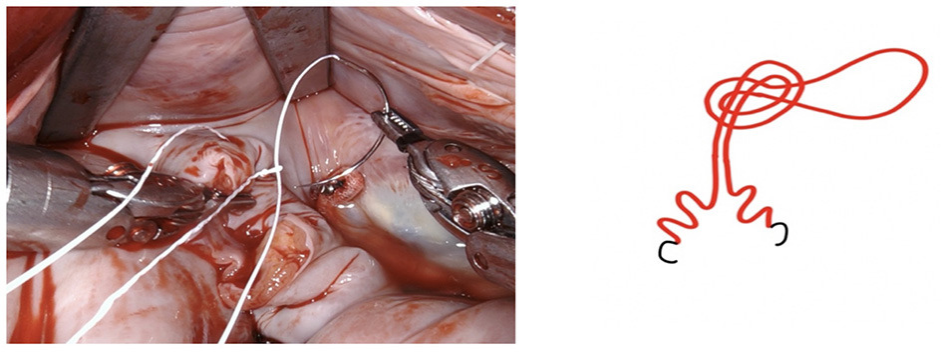
The suture is passed through the pre-fashioned loop, thus anchoring the starting point and sparing the time needed to create surgical knots.

**Figure 7:**
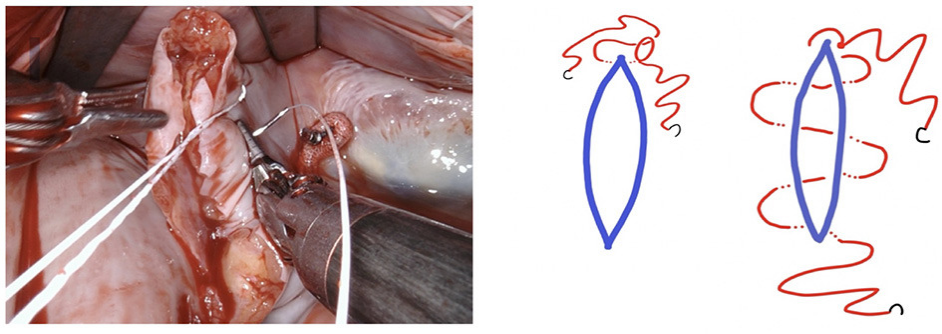
The first layer is a horizontal mattress suture, ensuring passage through all layers of the left atrial wall and pulling the free edge of the atrial defect inwards. This technique also facilitates a safe margin relating to the circumflex coronary artery.

**Figure 8:**
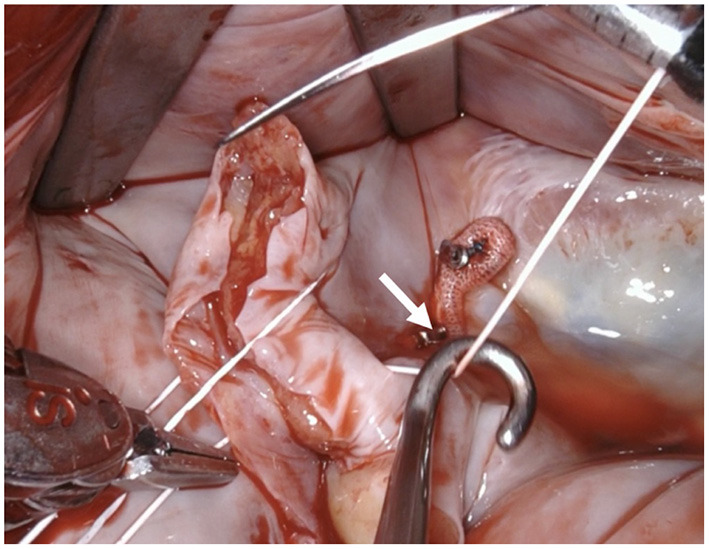
Tension of the suture is maintained by applying light traction using a suture hook (assistant, arrow).

**Figure 9:**
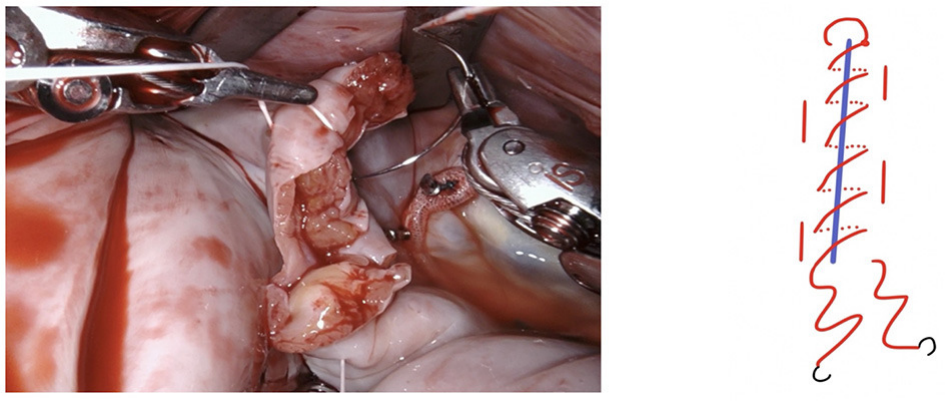
Using the remaining needle, a second layer of simple continuous suture is applied.

**Figure 10:**
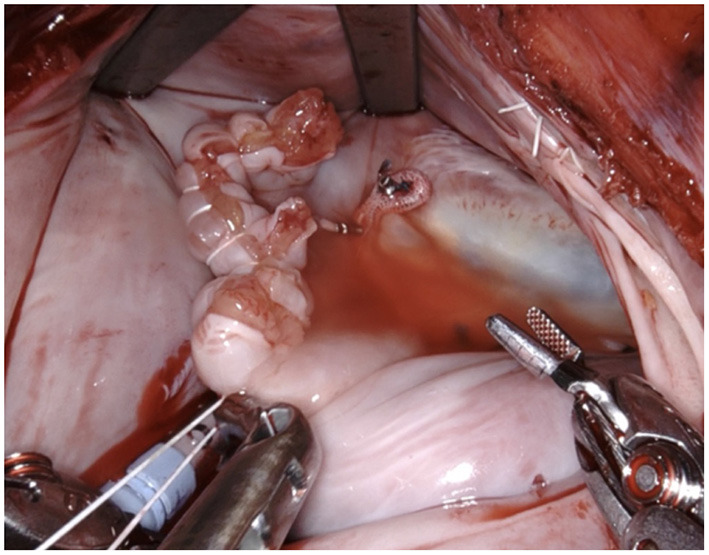
The needles are removed and the ends are tied off by the assistant using a knot pusher. Great care must be taken to apply adequate tension. In case of doubt an additional suture is placed preemptively as any problems with this suture cannot be handled once the left atrium is closed.

## Conclusions

The perfect and universally applicable surgical technique for LAA occlusion unfortunately does not exist, as it mainly depends on surgical access. Whereas external techniques have shown high success rates, purse-string and other suturing techniques applied from the inside carry a high risk of incomplete closure and residual perfusion (9). Our novel intra-atrial LAA cut-and-suture technique for robotic-assisted minimally invasive cardiac operations has proven to be safe, effective, and easily reproducible. Herein, we present a series of 20 patients without any procedure-related adverse events. So far, there is only one case report describing a similar technique (10). Other intra-atrial or LAA invagination techniques with ligation have been presented but not established into practice, potentially due to the increased thrombogenic risk of a remaining intra-atrial mass or stump (11, 12).

The technique itself, although first performed using a robotic surgical system, is easily applicable to any other type of minimally invasive surgery, e.g., mini-thoracotomy or thoracoscopic surgery. In our opinion, it has the potential to become standard-of-care for LAA closure from inside the heart because of its proven effectiveness and ease of implementation. Nonetheless, as seen in the photographic material, the quality of exposition and visualization of the relevant anatomy using robotic assistance is unparalleled. Stereoscopic vision and intuitive controls make the suture itself straightforward and technically less difficult than using conventional instruments. Meticulous suture technique is essential as any suture insufficiency can hardly be visualized and controlled from the outside, after reperfusion.

Problems commonly associated to the procedure of surgical LAA closure such as persistent blood flow into the LAA or incomplete occlusion are avoided, since the technique involves complete elimination of the LAA without a residual stump. Theoretically, the cessation of oral anticoagulation therapy after the standard recommended 3 months following surgery is feasible, ultimately reducing the rate of potentially life-threatening bleeding complications associated with chronic anticoagulant use. Nonetheless, a universally accepted standard practice regarding stopping anticoagulants after a given period of time does not exist, and the decision is made on an individual level by the physician.

## Limitations

Follow-up data are needed to prove long-term benefits of the technique.

## Data availability statement

The raw data supporting the conclusions of this article will be made available by the authors, without undue reservation.

## Ethics statement

Ethical review and approval was not required for the study on human participants in accordance with the local legislation and institutional requirements. Written informed consent for participation was not required for this study in accordance with the national legislation and the institutional requirements.

## Author contributions

Study conception and design: UF and NG. Data collection: MO and FH. Analysis and interpretation of results: MG, UF, MR, and NG. Draft manuscript preparation: MG and NG. Revised manuscript: UF, MO, FH, and MR. All authors reviewed and approved the final version of the manuscript. All authors agree to be accountable for the content of the work.
